# Evaluation of the K
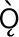
ts'iìhtła (“We Light the Fire”) Project: building resiliency and connections through strengths-based creative arts programming for Indigenous youth

**DOI:** 10.3402/ijch.v74.27672

**Published:** 2015-08-10

**Authors:** Sahar Fanian, Stephanie K. Young, Mason Mantla, Anita Daniels, Susan Chatwood

**Affiliations:** 1 Institute for Circumpolar Health Research, Yellowknife, Northwest Territories, Canada; 2 Social & Behavioral Health Science Division, Dalla Lana School of Public Health, University of Toronto, Toronto, Ontario, Canada; 3 Community Action Research Team, Tłįchǫ Government, Behchok 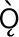 , Northwest Territories, Canada; 4 Department of Community Programs, Tłįchǫ Government, Behchok 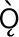 , Northwest Territories, Canada

**Keywords:** Dene, Indigenous, youth, evaluation, creative arts, resiliency, suicide prevention, health promotion, mixed methods, community-based research

## Abstract

**Background:**

The creative arts – music, film, visual arts, dance, theatre, spoken word, literature, among others – are gradually being recognised as effective health promotion tools to empower, engage and improve the health and well-being in Indigenous youth communities. Arts-based programming has also had positive impacts in promoting health, mental wellness and resiliency amongst youth. However, often times the impacts and successes of such programming are not formally reported on, as reflected by the paucity of evaluations and reports in the literature.

**Objective:**

The objective of this study was to evaluate a creative arts workshop for Tłįchǫ youth where youth explored critical community issues and found solutions together using the arts. We sought to identify the workshop’s areas of success and challenge. Ultimately, our goal is to develop a community-led, youth-driven model to strengthen resiliency through youth engagement in the arts in circumpolar regions.

**Design:**

Using a mixed-methods approach, we conducted observational field notes, focus groups, questionnaires, and reflective practice to evaluate the workshop. Four youth and five facilitators participated in this process overall.

**Results:**

Youth reported gaining confidence and new skills, both artistic and personal. Many youth found the workshop to be engaging, enjoyable and culturally relevant. Youth expressed an interest in continuing their involvement with the arts and spreading their messages through art to other youth and others in their communities.

**Conclusions:**

Engagement and participation in the arts have the potential to build resiliency, form relationships, and stimulate discussions for community change amongst youth living in the North.

The creative arts – music, film, visual arts, dance, theatre, spoken word, literature, among others – are gradually being used as a way to empower, engage and improve the health and well-being of communities (1, 2). As McDonald et al. (2) have shown, the arts can be a powerful tool for community building and organising because of its’ potential to bring a community together, bring attention to an issue, offer catharsis and to overcome language and cultural barriers. Interventions using the creative arts have also provided evidence that the arts can have positive public health implications. Stuckey and Nobel (1) conducted a review of the impacts of engagement with the arts on healing and wellness. They found that music, visual arts, movement-based creative expression and creative writing can have positive effects on healing and several health outcomes, such as anxiety and stress reduction, mood improvement, increased self-awareness, self-esteem, self-worth and identity, ability to make meaning and to cope with challenging experiences.

The arts also play a significant role in Indigenous cultures around the world (3). The Aboriginal Healing Foundation (AHF) has produced a report outlining the ways in which traditional culture and creative arts are being used in community-based programmes to facilitate healing and to further build collective strength among First Nations, Inuit and Metis peoples in Canada (4). The AHF provides a 3-way framework describing the interrelationship between creative arts, culture and traditional healing and evidence that when given the freedom to choose, many community-based healing programmes overwhelmingly include the arts (4).

Arts-based interventions have been particularly successful among youth (4–6) and are increasingly being used to address several challenges faced by Indigenous youth (5). There are a number of community-led initiatives using arts-based approach in Indigenous communities. For example, Blueprint for Life is a programme that uses hip hop to facilitate healing, build confidence, self-identity and self-esteem and foster strong relationships among Inuit youth in the Arts (7). FOXY is an arts-based participatory action research project using creative arts to empower teenage girls with sexual health decision-making (5). Similarly, Taking Action uses creative arts as a tool for HIV prevention and awareness by training Indigenous youth to become leaders in their communities (6). These initiatives have highlighted all the ways in which the arts can promote dialogue and raise awareness on various health issues, facilitate healing, build capacity, skills and confidence among youth, and strengthen connections between youth and their communities at large.

Many of the outcomes described above, such as confidence, self-esteem, self-identity, healthy relationships, skill-building and empowerment, are important factors in building resiliency (8). In the context of exposure to significant adversity, resilience can be understood as “both the capacity of individuals to navigate their way to the psychological, social, cultural, and physical resources that sustain their well-being, and their capacity individually and collectively to negotiate for these resources to be provided in culturally meaningful ways” (8). Although engaging in the creative arts has been linked to positive effects on health in a variety of contexts (1), little has been written about the potential role of the arts in building resiliency. Thus, the intent of this article is to share the outcomes of an evaluation of K
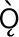
ts'iìhtła, a creative arts workshop for Tłįchǫ youth, to contribute to the growing body of literature linking the arts to resiliency among youth.

## The K
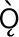
ts'iìhtła project: an overview

Using the arts as a vehicle for empowering and building resiliency among youth was identified as an important potential strategy by the authors of this article and youth who attended a suicide prevention workshop in May of 2014 in Toronto. During the workshop, one of the authors, MM, shared a film he directed in his community on the topic of youth suicide. The film was created with the goal of engaging youth in his community and raising awareness about suicide in the community. Other Indigenous youth at the workshop also spoke about their connections with different forms of art, such as music and the spoken word. The idea behind K
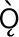
ts'iìhtła was generated during a breakout session where the authors and several contributors discussed the role art could play in building youth resiliency. At its inception, K
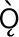
ts'iìhtła was envisioned as a community-based and youth-led project.

Since MM was a member of the Tłįchǫ Community Action Research Team (CART) in the Northwest Territories (NWT), the project was built on existing capacity and established relationships with the community of Behchok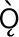, NWT. Since 2009, CART was established with the goal of turning research into action and promoting health among children and youth in the Tłįchǫ region. They evaluate community issues through participatory research-based programming and arts-based approaches to research. Guided by the Healing Wind Advisory Committee, a committee of Elders and community representatives, CART integrates knowledge of Tłįchǫ values and beliefs into their work at all stages of programming and research processes. Over the years, CART have created a strong foundation in using arts-based participatory methods to engage youth voices, build capacity and create healthy and supportive youth communities (9, 10).

The K
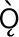
ts'iìhtła (“We Light the Fire”) project was a 5-day creative arts and music workshop for youth which ran from August 11 to 15th, 2014, in the community of Behchok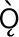, NWT, with the aim of empowering youth to explore critical issues facing their community and their lives and to find solutions together using the arts. The project was hosted by the Tłįchǫ CART in Behchok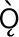 and originally stemmed from the need to address high rates of suicide in northern Aboriginal communities. While suicide prevention among youth was the original impetus for the project, the workshop took a strengths-based approach by focusing on empowering and building capacity amongst youth using the creative arts.

K
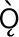
ts'iìhtła aimed to build resilience by ensuring that youth have ownership of the workshop and its outcomes. Youth were given the opportunity to identify topics or issues they wanted to address based on what was important to them. The project also focused on building a culturally relevant space for youth to engage with the creative arts by celebrating Tłįchǫ culture and offering workshops led by Tłįchǫ creative mentors. The Tłįchǫ CART recruited Indigenous facilitators to deliver the programming, 3 of the 5 facilitators were Tłįchǫ and from Behchok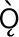. During the workshop, participants were encouraged to share their stories and took leadership roles in directing their focus for the creative art projects. Facilitators grounded their workshops on the principal of reciprocity and shared learning and dedicated time to share their personal stories with the participants. In doing so, this workshop created an open and safe space to share stories and promoted cultural relevancy through youth-identified topics.

Additionally, K
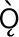
ts'iìhtła responded to the need to improve availability, access, cultural-safety, quality and continuity of mental wellness and addictions programming in the NWT, a long-standing priority of the Government of the Northwest Territories (GNWT). Recently, the dearth of prevention and early intervention programmes targeting children and youth mental health and addictions issues was identified as one of the most critical and persistent service gaps in the NWT (7). While supporting youth-led strengths-based mental wellness programming, K
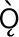
ts'iìhtła also shared many of the same community wellness goals as the Tłįchǫ Community Services Agency and those of the GNWT's Mental Health and Addictions Action Plan (7). These goals include engaging youth communities in discussion of mental health and addictions challenges, developing approaches to share best practices among communities and regions, developing strategic communication plans to deliver ongoing mental health and addictions, stigma, suicide prevention and resiliency-related campaigns, and building community capacity for community-led initiatives to support mental wellness (7).

**Fig. 1:**
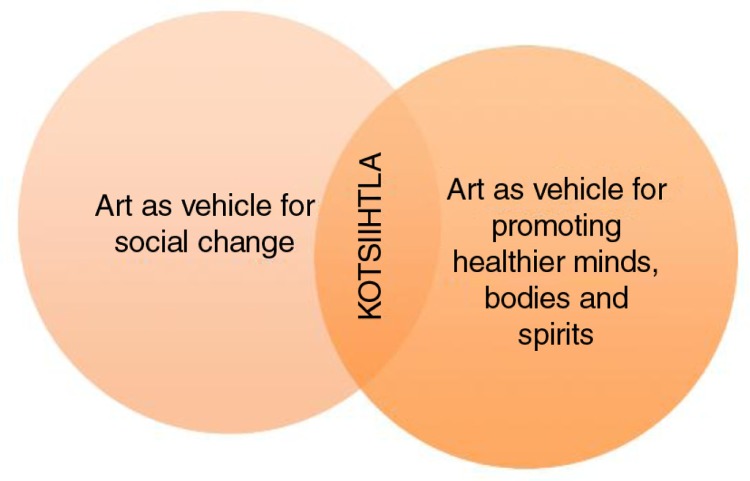
K
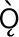
ts'iìhtła goals.

Thus, the overall goals of K
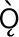
ts'iìhtła were twofold: to engage and empower youth to explore critical issues in their communities and lives and to find solutions together using creative arts (*art as vehicle for social change*), and to build resiliency amongst youth and promote healthy minds, bodies and spirits through the arts (*art as vehicle for promoting healthier youth and communities*). The objectives included: (a) building confidence and personal/artistic skills among youth participants, (b) connecting youth with one another and to positive role models and (c) demonstrating to youth how art can be a way to express oneself and to deal with various issues in our lives and communities (Fig. 1).

In doing so, the project aimed to:

- Build on the collective and individual strengths of youth
- Provide an opportunity for youth to engage in the arts and to voice their thoughts/beliefs/emotions using the arts
- Develop youths’ artistic and personal skills, self-confidence, self-expression, sense of identity and overall well-being
- Connect youth with positive role models and with one another

Values of K
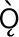
ts'iìhtła:

- Community-based
- Youth-friendly
- Strengths-based approach
- Rooted in Tłįchǫ values and traditions
- Respect and creating safe spaces
- Ownership, control, access and possession (OCAP)

The objectives of K
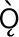
ts'iìhtła:

- To provide a mentorship opportunity for youth to learn artistic and personal skills from local, Indigenous artists and from their peers
- To develop youths’ resiliency, confidence, self-expression and skill-set through music and creative arts
- To provide resources and a safe space for youth to discuss pertinent issues in their communities and lives and to find solutions together using the arts
- To share youth participants’ artwork and messages with other youth in their community and around the world, if they desired
- To develop a community-led, youth-driven model for continued youth engagement in the arts in Behchok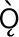 and explore implications for circumpolar regions.

## Current study

The goal of this study is to evaluate K
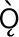
ts'iìhtła and identify successes, challenges and unexpected outcomes that occurred during the workshop. The present study describes our evaluation method, key findings and lessons learned. Additionally, this study has catalysed the development of community-oriented knowledge translation resources and toolkits to support creative arts programming for Indigenous youth.

## Method

### Partnership

The evaluation of K
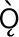
ts'iìhtła was a collaborative, community-based research project led through a partnership between the Tłįchǫ CART and the Institute for Circumpolar Health (ICHR). Two research assistants from ICHR and the social programme coordinator with CART developed the evaluation framework and tools and assisted with data analysis. The research assistants from ICHR supported workshop planning, data collection and analysis. All participants within the partnership participated in the authorship of the article.

### Participants

**Fig. 2:**
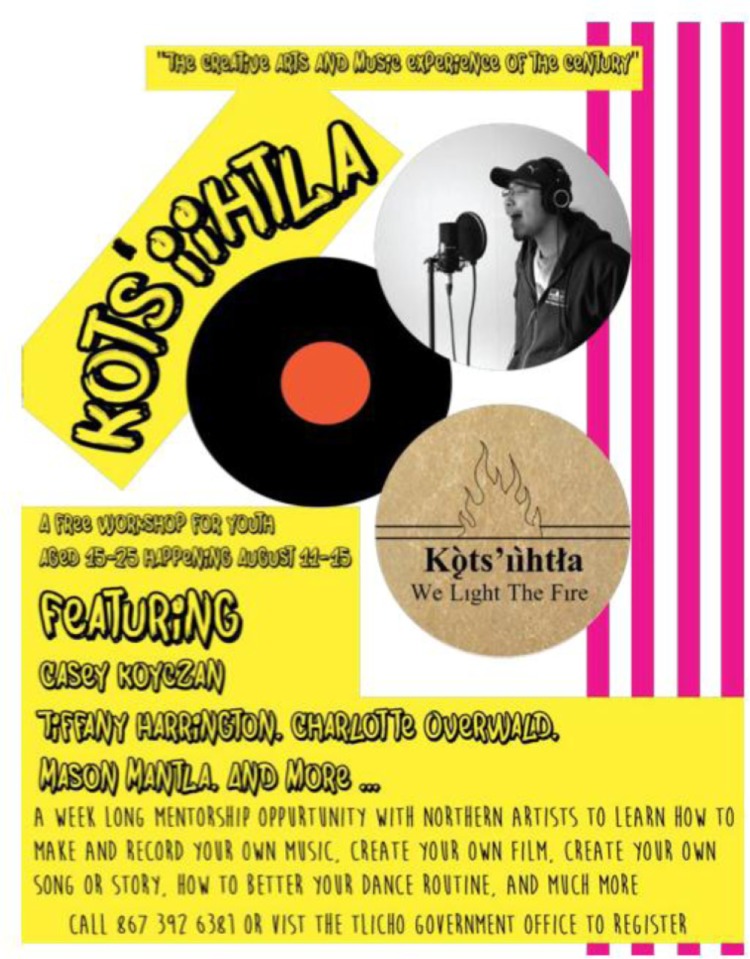
Promotional poster for the K
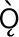
ts'iìhtła Workshop.

Youth were recruited to participate in the workshop through many ways: Tłįchǫ CART staff, in-person presentations, promotional posters (Fig. 2), word of mouth, radio and social media. Nine youth participated in the workshop overall, with an average of 5–6 youth per day. Several of the same youth came consistently for all 5 days, and other youth were introduced to the programme mid-week from their friends who were already participating. All youth were Tłįchǫ and from the community of Behchok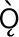, NT. The youths’ ages ranged from 13 to 22; there were 4 females and 5 males overall. Five youth were unavailable to complete the youth feedback questionnaire because they did not attend the last day of the workshop. Four youth completed the questionnaire, whose ages ranged from 14 to 22; there were 2 females and 2 males overall.

Five Indigenous artist facilitators delivered the creative arts programming. Each facilitator brought a diverse set of artistic backgrounds and expertise, which included spoken word, sound production and design, film, photography, multimedia arts, jewellery making and visual arts. All 5 facilitators participated in the evaluation.

### Evaluation framework

An evaluation framework was developed to identify the successes and challenges associated with the programme, ways, outcomes and impacts can be increased, and to understand the benefits participants received from being involved in the creative arts and collaborative projects. These evaluation findings are intended for use by community governments, youth organisations, and programme coordinators to provide insight and improve future youth-driven, strengths-based and creative-arts programming. Correspondingly, the evaluation framework and tools aimed to capture both process (evaluating the activities and events that occur as part of implementation, what are the strengths and weaknesses of day-to-day operations, how can these processes be improved) and outcome (evaluating the extent to which programme goals and outcomes have been obtained) objectives. In doing so, the framework details the evaluation objectives, respondent(s), indicators, methods and timing of data collection. The evaluation framework is described in detail in Appendix A.

### Data collection

Programme evaluation was integrated throughout the entire workshop. Observational data were collected every day by recording youth attendance numbers, different activities youth engaged in that day and comments from conversations facilitators and research staff had with different youth about their art and the art mediums they were working with. At the end of each day, a focus group was conducted to explore youth and facilitators impressions about the day and their experiences. Field notes were taken, and at the end of each day facilitators wrote a reflection. Two sets of questionnaires were developed, one for youth participants and one for artist facilitators. Both questionnaires were administered and collected at the end of the workshop. The youth questionnaire assessed 6 main areas: (a) recruitment, (b) satisfaction; (c) areas of success and areas in need of improvement; (d) cultural relevancy and appropriateness; (e) personal impact and (f) desire to continue engaging in the arts. The facilitator questionnaire assessed 6 main areas: (a) satisfaction; (b) areas of success and areas in need of improvement; (c) challenges encountered; (d) experience working with youth; (e) overall impressions and (f) continuing youth engagement and capacity building in the arts. Sample questions from the youth and facilitator questionnaire can be found in Tables I and II, respectively. Finally, practice and field notes were incorporated to support the monitoring and evaluation of the workshop and contribute to organisational learning and capacity development.

**Table I:** Sample questions from youth participant feedback questionnaire

Evaluation objective	Sample questions
To understand what elements of the workshop could be improved	Did the workshop meet your expectations? Why or why not? What parts of the workshop did you dislike or feel could be improved? Why? What would you have liked to learn more about?
To understand whether the workshop had a positive impact on youth	Did you find the workshop fun? How has this workshop impacted you? Would you do this workshop again in the future? Why? Would you like to stay connected with the people in the workshop? What do you feel you have gained from the workshop?
To understand appropriateness of workshop	Did you feel comfortable working and speaking with the facilitators? The other youth? Why or why not? Did you feel the workshop was culturally relevant?
To understand youths’ interest in continuing art initiatives and sharing of their art	Would you like to share your artwork and music with others? If so how?

**Table II:** Sample questions from the facilitator feedback questionnaire

Evaluation objective	Sample questions
To understand what elements of the workshop were successful and what could be improved	What do you consider some successful components of the workshop? Why? How do you feel the workshop could be improved? What challenges did you encounter during the workshop? How did you overcome them?
To understand whether the workshop had a positive impact on youth	From your perspective, what impact did the workshop have on the youth participants? What do you feel was accomplished or gained from the workshop?
To understand ways to continue arts-programming and share the art beyond the scope of this workshop	Would you help with this workshop again in the future? If so, why and in what capacity? What are some ways we can help the youth participants to continue to use the arts, and reach out to other youth using arts/music

### Data analysis

All data, including the participant and facilitator questionnaires, focus group notes, reflective practice notes and field notes, were de-identified, cleaned, transcribed and entered into Microsoft Word. Data were analysed using an adapted version of the triangulation protocol described by Farmer et al. (11). Two researchers conducted a separate qualitative thematic analysis of each data source respectively. Three types of triangulation were used to analyse the data:

- Multiple Investigators (“involving 2 or more researchers in the analysis”): Two researchers independently analysed the data sources and compared results.
- Methodical (“using more than one research method or data collection technique”): Results were analysed from various methods of data collection (questionnaires, focus group notes, reflective practice notes and field notes)
- Data Source (“using multiple data sources or respondent groups”): Three perspectives were represented in the data analysis (programme coordinators, facilitators and participants).

A “convergence-coding matrix” was created to display the themes that emerged from each data source on 1 main page using Microsoft Excel. Findings were coded for agreement, partial agreement, silence or dissonance. Two researchers then assessed the findings for convergence, completeness and compared the level of agreement between researchers. The emerging themes were discussed and agreed upon by 2 researchers. One researcher structured the main findings into groups, which were reviewed and agreed upon by all authors.

## Results

The data were compiled and grouped into the perspectives of the youth, facilitators and art outputs.

### Youth perspectives

Youth were recruited to join the workshop through a variety of strategies. The youth participants reported hearing about the workshop either through posters, word of mouth or speaking directly with CART members. The youth participants gave different reasons for wanting to join K
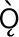
ts'iìhtła including, an interest in learning more about the arts, like film and media, and to improve their artistic skills. Others gave reasons such as having friends who joined, wanting to get out of the house and do something or wanting to have fun. One youth participant wrote, “I joined because I wanted something to do, to get out of the house and participate.” The majority of youth participants said that the workshop met their expectations; however, some commented that they wished more people joined. When asked what components of the workshop they liked, youth participants wrote about having had the chance to learn and engage with different forms of art, like photography, painting, singing, filming, working on the music video, etc., as assets. They commented on how it was fun to take part in and new for them. When asked about components of the workshop they did not like, responses were minimal. However, some identified wanting to see more youth involved in the workshop. Many of the youth participants also said that they wanted to pursuit arts after completion of the workshop. Some were interested in learning more about or continuing street art, while 3 out of the 4 youth who responded to the survey wanted to pursue film. All youth participants also found the workshop to be fun and engaging, citing being provided with food, meeting new people and avoiding boredom as reasons for why they thought the workshop was worthwhile. Three out of four participants said that they wanted to take part in more workshops like K
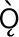
ts'iìhtła and suggested having art and music programmes after school in the community. Most also found the workshop to be culturally relevant and felt comfortable working with the facilitators. Some of the female youth participants, however, pointed out that they only felt comfortable working with the female facilitators. All of the youth said that they gained something positive from the workshop, responses were mainly from making new friends and learning new art skills. All of the youth expressed a strong desire to share and disseminate their artwork.

### Facilitator perspectives

Facilitators completed an open-ended questionnaire and took part in debrief sessions following each day of the workshop. Qualitative findings from the facilitators provided useful feedback on the process and functioning of the workshop as a whole, as well as its success and challenges. While the facilitators found the workshop to be successful in achieving its goals, in the products made and importantly, in the relationships formed, many identified components of the workshop that could be improved for the future. Facilitators and youth participants both said that having more youth participants in attendance and a balanced student: facilitator ratio would have strengthened the success of the workshop. Facilitators also suggested focusing in on 1 or 2 art forms and a couple of finished products, which could have allowed for a more in-depth learning experience and an easier time discussing and producing a final piece of art. More structure and pre-planning sessions also could have helped with this. A couple of the facilitators also suggested establishing a theme for the final art products on the first day; however, while this may have helped focus the workshop activities, youth participants may not have felt comfortable on the first day discussing certain sensitive topics. Other suggestions included training facilitators on the local history and culture in preparation for the workshop and also reducing the amount of music and recording equipment so as not to intimidate the youth from trying it. To further improve the workshop by making the activities even more youth and community led, facilitators recommend sharing the art products at a community event (if the youth express an interest to do so and feel it is appropriate). Furthermore, they stressed the importance of ensuring that youth have access to supplies, necessary equipment and space upon completion of the workshop so that youth can continue to develop their interests in the arts of their choice.

Overall, facilitators found the workshop to be a positive experience. They commented that not only did participating in the workshop build confidence in themselves in their new role as a facilitator, they also recognised striking changes in confidence among the youth participants. One facilitator wrote, “the workshop seemed to increase the students’ confidence in the art forms and overall personality, as many of them came out of their shell by the end of the week.” In their conversations with the youth participants, facilitators also found that youth commented on how the workshop taught them new skills, and provided new possibilities and opportunities for them to explore the arts in the future. Finally, facilitators talked about the relationships formed between themselves and the youth participants as being one of the most positive components of the workshop.

### Art products

**Fig. 3:**
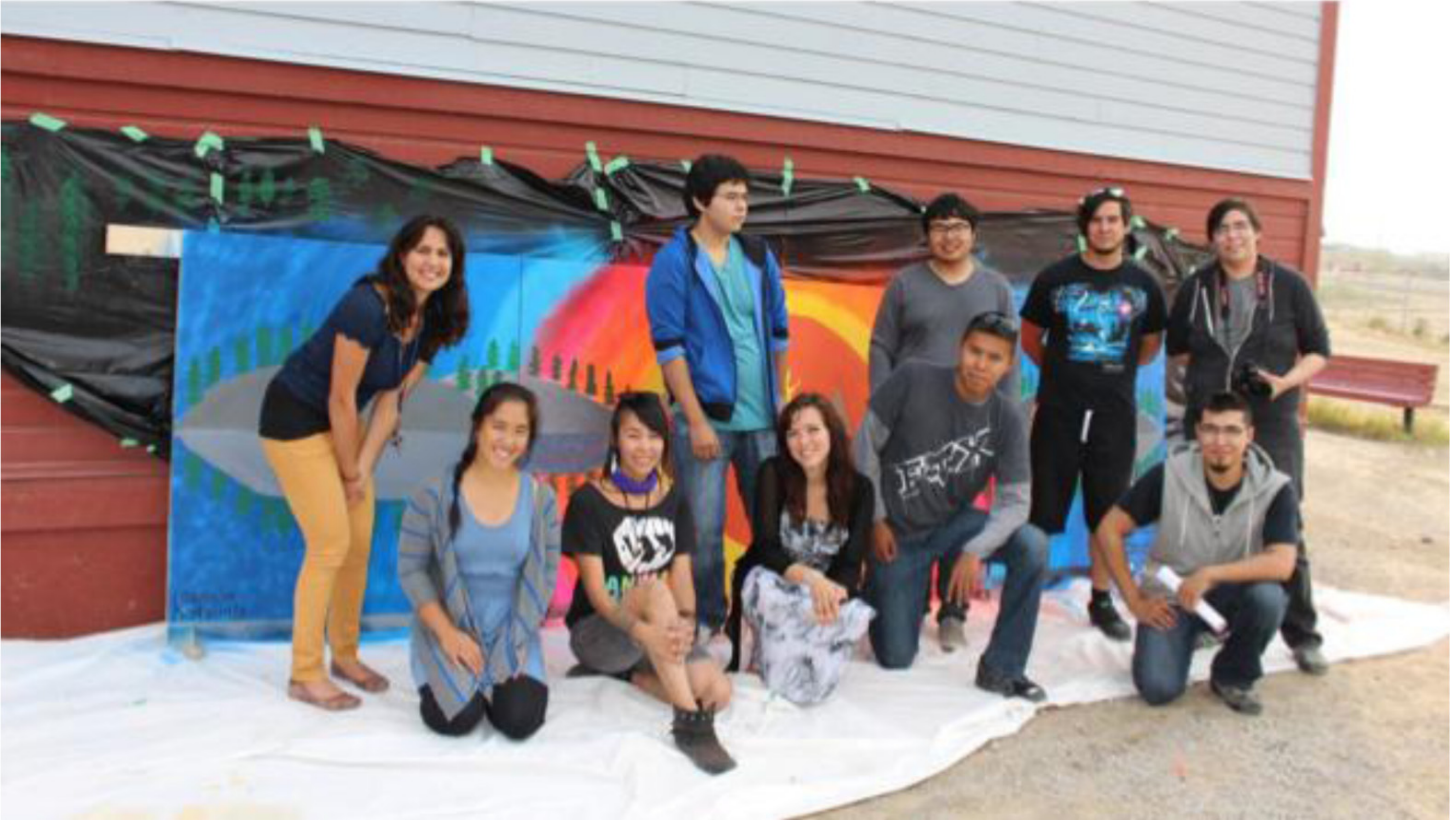
Workshop facilitators on the last day of the workshop in front of the collaborative mural that the youth created with workshop facilitator Charlotte Overvold. Photo credit: Sahar Fanian.

By the end of the 5-day workshop, youth participants and facilitators collaboratively created a mural (Fig. 3), music video and short film, each representing different themes, challenges and hopes discussed by the youth. The process involved brainstorming all together or in small groups what they wanted represented in their art and how they wanted it represented. The process of brainstorming brought up significant points of discussion for the youth participants, allowing them to express their concerns, hopes and visions for their community and lives. Pictures from the music video production and lyrics for the music video can be found in Appendix B.

## Discussion

Overall, we found that youth participants had a positive experience participating in K
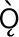
ts'iìhtła because of 4 main factors: they developed new skills, had positive interactions with facilitators, found the workshop to be culturally relevant and enjoyable and used the arts to talk about community issues and visions for change. All of the youth participants identified having gained new skills in the arts and all expressed an interest in continuing and building on their artistic skills after completion of the workshop. Youth gained skills in visual arts, music and music production, film-making, photography, song writing and spoken word.

Facilitators also found that several of the youth showed a noticeable increase in confidence over the course of the workshop, with many transitioning from very shy to confident by the end of the week. A second important success of K
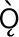
ts'iìhtła, as identified by participants and facilitators, was the positive interactions and relationships formed between the youth, and between the youth and facilitators. Many of the youth participants had the opportunity to spend one-on-one time with the facilitators, giving them a chance to build on their artistic skills but also to share their thoughts, concerns and aspirations and to hear facilitators’ advice and personal stories. There were opportunities for bonding, learning and sharing between the youth participants and facilitators. Youth participants also found the workshop to be culturally relevant, because it was based on Tłįchǫ values and traditions (11) of “being strong like 2 people,” where “young people are strong physically and spiritually; determined yet cautious; able to read the environment for survival,” “work as part of a community,” and share and learn on the land (p. 13). Youth participants and facilitators also saw the workshop to be successful because it was led by Indigenous youth artists, some of whom were from the community of Behchok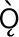.

All of the participants found that the workshop to be fun and enjoyable, and many said that they looked forward to coming to the workshop each day. We observed that youth took pride in their individual and collective artwork, such as the music video, short film and mural, and expressed an interest in sharing their artistic products with other youth and others in their community. Finally, the workshop functioned as a catalyst for discussing issues and visions in their community and lives. One-on-one and in groups over the course of the workshop, the youth participants began to share about challenges such as alcohol use, cyber bullying and suicide and employment, as well as positive aspects of their community and visions for their own and collective future through the artwork and in conversations. This was displayed through their collaborative music video and mural, which participants hoped others in the community would see and would stimulate conversation about challenges, strengths and changes in the community as a creative and inspiring method of knowledge translating and sharing.

While we are still in the process of arranging for youth participants to display and discuss their work at the upcoming Tłįchǫ CART's Annual Youth Conference, our experience from K
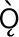
ts'iìhtła has shown us that the arts have the potential to build resiliency, form relationships and stimulate discussions for community change amongst youth in the North. We also created a community-friendly version of this report to share with Tłįchǫ communities and others interested in developing or running community-based creative arts programming for youth in their own community. Positioning the project within the community's health authority and building off the established strengths of CART promoted project sustainability.

### Lessons learned

- Flexibility is essential. Each day the structure or agenda for the day should be re-evaluated and adjusted accordingly depending on the number of youth participants, available facilitators, youths’ interests, space and facility restrictions and other circumstances.
- It is important to balance structure and non-structured time throughout the workshop, providing ample opportunities for youth to collaborate and engage in a new art form art, while also providing the space and time for youth to autonomously choose what suits their needs.
- It is as important to facilitate group building and cohesiveness as it is to facilitate individual growth. However, group activities, such as sharing circles, cannot be “forced,” they often happen organically over the week, as participants become more comfortable with one another.
- It is important to create a safe space by establishing ground rules as a group. This gives youth participants the opportunity to share, if they choose to, and feel safe about it while also reminding the group to respect the confidentiality of others.
- Youth participants should have ownership over their art. They choose what, how, where and when they wish to showcase their artwork.
- The workshop should have ample opportunities for youth collaboration and sharing of talents and skills.
- Artist facilitators should not only be skilled in their respective art medium but also have some shared ground or experience with the youth.
- Depending on the number of youth and their interests, it may be worth selecting only a few types of art that met the needs and interests of youth participants rather than having too many activities going on.
- Ensure there is an appropriate ratio of facilitators to youth (approximately 1 facilitator per 3–4 youth).
- Create a safe, culturally relevant, respectful and creative environment for youth participants.
- It is important to determine what the workshop goals and objectives are and whether the project will be “process-driven” (the process of making the art has benefits and is valuable in and of itself) or “product-driven” (product has a value, e.g. mural will be put up in the community or music video will be shared with other youth online).

## Limitations

There are a number of limitations to our results. First, K
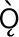
ts'iìhtła participants were self-selected. Only youth who were interested in creative arts or were available for the week would sign up to participate. Only 4 youth completed the full questionnaire, limiting our ability to generalise results to the whole group. With such a small sample size, it is difficult to make any firm conclusions. However, we did get input from youth through other means from the debrief and focus group sessions, from observational data and field notes, as well as from all facilitators, which has provided us with more data than just the questionnaires. Second, youth who attended the workshop in some cases were variable, with some youth dropping out within the first couple days while others joined later in the week. As a result, we do not have consistent data from all youth and are missing data from those who dropped out early as to the reasons why they did not continue. Although others in the group have informed us that work schedules, particularly mining shift work, may be responsible for the gaps in attendance.

## Next steps

Youth expressed an interest in continuing their engagement with the arts and desire to share their messages with other youth and others in their community through their artwork. They shared their views that the music video and film can be used as a way to connect with youth across the world and to establish shared experiences, support and empowerment. Participants had a strong desire to share their work online through well-known media and social-media websites, as well as to share their work with the community as a whole through a community showcase or event. Thus, we are presently working with the youth and some key partners to discuss how their artistic achievements can be shared with others. In particular, some of the art products will be featured at the upcoming Tłįchǫ CART's Annual Youth Conference. We are also working with community partners to see how we can integrate art activities into existing community initiatives. The goal of K
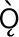
ts'iìhtła is to be a community-led and youth-driven initiative to continue supporting creative art opportunities for youth in circumpolar communities.

## Conclusion

Evaluation results from K
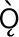
ts'iìhtła, a pilot creative arts and music workshop held in the summer of 2014 for youth in Behchok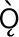, have shown the potential for art to be used as a medium for building resiliency, forming positive relationships and stimulating discussions on community change among Tłįchǫ youth and shows potential for Indigenous youth in Northern Canada. As this was a pilot community project with a small number of participants, we encourage others to continue examining the role of creative arts as a tool for social change among youth communities and share findings. By substantiating the existing literature on arts-based health promotion programmes for youth, we hope that these results can be used to support the sustainability of arts-based programmes for Indigenous youth. Next steps will involve helping to build a knowledge exchange platform for K
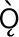
ts'iìhtła youth to share their artwork with others with the hope to stimulate youth-led discussions within and across communities in the circumpolar regions.

## Figures and Tables

**Figure:**
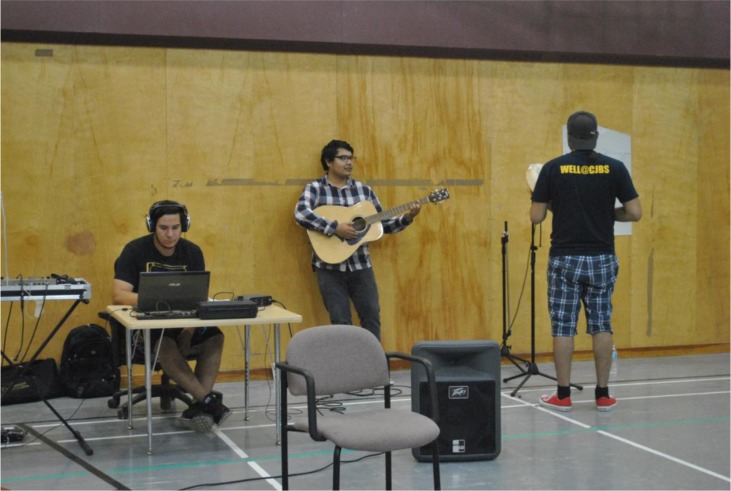
Recording components of the music for “We Write the Future”. Photo credit: Sahar Fanian.

**Figure:**
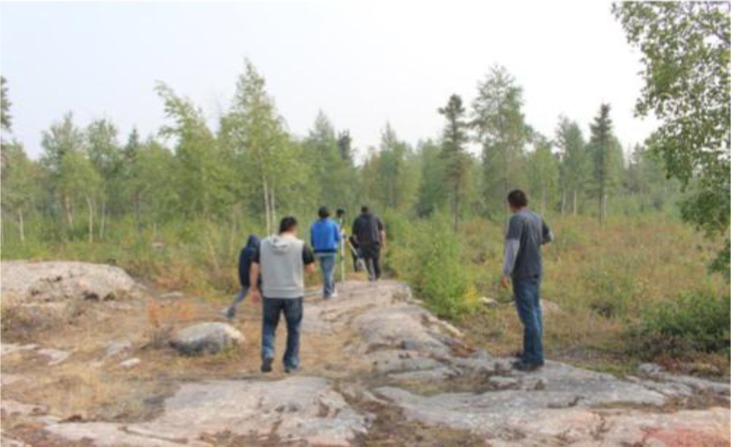
Youth and facilitators in action, shooting scenes for the music video *We Write the Future*. Photo credit: Sahar Fanian.

## Appendix

**Table:** Appendix A: Evaluation framework

Evaluation objectives	Target population/respondents	Indicators	Data collection methods	Timing of data collection
To understand how youth heard about Kts'iìhtła and why they decided to join	Youth participants	Successful method(s) of recruitment; youths’ initial motivation for participating	Questionnaire Focus groups	Last day of workshop At the end of each day
To understand what elements of the workshop youth and facilitators liked and what elements they felt could be improved	Youth participants and facilitators	Number of participants Impressions of the workshop day-to-day activities; overall satisfaction with workshop; elements liked/disliked; level of engagement during each activity	Overall attendance and attendance at specific activities Questionnaire Focus groups, debriefing sessions, reflective practice notes, field notes	Attendance each day Last day of workshop Throughout workshop
To understand whether the workshop was culturally relevant	Youth participants and facilitators	Self-assessed feedback on cultural relevancy by youth participants and facilitators; Protocols (asking for permission and informed consent, reciprocity), Partnerships (relational practice and collaborative problem solving), Personal knowledge (self-location and sharing of personal experience), Process (negotiating goals and activities), Positive purpose (building on strengths, meaningful experience for participants)	Questionnaire Focus groups, debriefing sessions, reflective practice notes	Throughout the workshop
To understand appropriateness of workshop	Youth participants and facilitators	Level of comfort between facilitators and youth; level of comfort amongst youth participants	Questionnaire Focus groups, debriefing sessions, reflective practice notes, and field notes	Last day of workshop Throughout workshop
To understand youths’ and facilitators’ intentions to engage with the arts and/or future art workshops after this pilot workshop	Youth participants and facilitators	Future interest and intentions to be involved in the arts and/or Kts'iìhtła art initiatives	Questionnaire Focus groups, debriefing sessions, reflective practice notes	Last day of workshop Throughout workshop
To understand whether the workshop had a positive impact on youth	Youth participants and facilitators	Level of positive influence of the programme; Skills, confidence, self-esteem, positive relationships, etc. gained; Self-reported learning new skills related to creative arts; Self-reported positive influence from opportunities to collaborate with other youth/Aboriginal artists	Focus groups, debriefing sessions, field notes, reflective practice notes Questionnaire	Throughout workshop Last day of workshop
To understand youths’ interest in continuing art initiatives and sharing of their art beyond the scope of this workshop	Youth participants and facilitators	Interest and level of enthusiasm for sharing work and continuing art initiatives like Kts'iìhtła	Questionnaire and focus groups	Last day of workshop

## Appendix B: Pictures of the music video production and lyrics for the music video *We Write the Future*

**Table:**

Lyrics to “We Write the Future” Chorus: We write the future x2 I'm dealin’ with my demons on the daily but it's not enough to drown me in this storm when the waters in the sea get rough Lookin’ down below I'm searching’ for a dash of clarity But smouldering’ in flames my fears clash with sincerity Chorus We compose the future but at times we stuck in writers block Obstacles be in my way when I'm racin’ against the clock Darkness breathing down my neck Close enough for me to touch Reachin’ out for courage cause alone this is a bit too much. A thousand years I've been frozen in perpetual paralysis But my visions become clearer under closer analysis I realize I'm the only obstacle in my way so now listen to me closely to hear what I'm bout to say Chorus
